# Uterine sarcoma: A 10-year retrospective analysis of clinicopathological and prognostic factors of surgically treated cases

**DOI:** 10.3332/ecancer.2026.2055

**Published:** 2026-01-09

**Authors:** Japhia David, Ruchi Arora, Abhilash Vasanth, Kaustubh Burde, Bijal Patel, Chetana Parekh

**Affiliations:** Department of Gynaecological Oncology, The Gujarat Cancer and Research Institute, Ahmedabad, Gujarat, India

**Keywords:** uterine sarcoma, overall survival, disease-free survival, recurrence, prognosis

## Abstract

**Background:**

This study aims to analyse the clinicopathological features of uterine sarcoma (US) and its impact on the survival outcomes.

**Methods:**

This is a retrospective observational study including 85 patients of histologically proven US, surgically treated during the period of January 2010 to December 2019. The patients were staged according to the FIGO staging system. Surgery was the mainstay of management with adjuvant treatment administered based on the stage and histopathological subtype. Descriptive analysis of the sociodemographic and clinicopathological features was done and survival analysis was done using Kaplan-Meier method.

**Results:**

The mean age of presentation in our study was 50.8 years for uterine leiomyosarcoma (ULMS), 45.5 years for low grade endometrial stromal sarcoma (LGESS), 48.1 years for high grade endometrial stromal sarcoma (HGESS) and 49.6 years in the OTHERS group. The majority were diagnosed in stage I (ULMS = 37.6%, LGESS = 16.5%, HGESS = 4.7%; OTHERS = 4.7%). The overall recurrence rate was 56.5%. 5-year overall survival (OS) and disease-free survival (DFS) rate of adenosarcoma (AS) was 100%, respectively. LGESS showed a 5-year OS and DFS rate of 86.3% and 60.5%, respectively. The 5-year OS and DFS rate of ULMS was calculated to be 77.7% and 39.9%, respectively. The 3-year OS and DFS of HGESS has been calculated to be 58.3% and 22.2%, respectively. Significance of serum lactate dehydrogenase value, stage of the tumour and the nature of adjuvant treatment were demonstrated for recurrence.

**Conclusion:**

US tend to recur. However, AS and LGESS show better survival outcomes as per our study. A proper workup with intervention including appropriate adjuvant treatment would help to mitigate the severity of the disease.

## Introduction

Uterine sarcomas (US) constitute a rare group of gynaecological malignancies arising from the uterine mesenchymal cells [1]. They account for about 3% of all uterine malignancies and 1% of all female tract malignancies [1, 2]. Uterine Leiomyosarcoma (ULMS) is identified as the most frequent pathological subtype as per previous studies with an incidence of 0.86 per 1 lakh women [3, 4]. Other often-identified subtypes include, endometrial stromal sarcoma (ESS) further subclassified as high grade ESS (HGESS) and low grade ESS (LGESS) and undifferentiated US (UUS). A few uncommon subtypes include adenosarcoma (AS), perivascular epithelioid tumours and uterine tumours resembling ovarian sex cord stromal tumours. USs are considered to be aggressive tumours with poor prognoses [5]. However, the prognosis depends largely upon the histopathological type [6]. Surgery remains as the mainstay of management depending on the stage [7]. This study aims to analyse the clinicopathological features of US and its impact on the survival of patients who had undergone surgery.

## Material and methods

This is a retrospective single institutional observational study done at The Gujarat Cancer and Research Institute, Ahmedabad, India including the period of January 2010 to December 2019. The data of 85 patients surgically treated during the study period were recruited for the study. Ethical clearance was obtained from the institutional ethics committee of our institute vide number IRC/2025/P-58.

This study included all the patients with histopathologically proven diagnoses of US either primarily diagnosed and treated at our institute or treated outside but had their histopathological slide reviewed at out institute and continued strict follow-up at our institute. Follow up included physical visits and telephonic conversations regarding the survival status. The patients with improper data and improper follow-up were excluded from the study.

The patients were staged according to the FIGO staging (2014). Surgery was the mainstay of management. Surgery included Total Abdominal Hysterectomy (TH) with bilateral or unilateral salpingo-oophorectomy (USO/BSO). Omentectomy and lymph node dissection (LND) were done in a few earlier cases. Adjuvant treatment in the form of chemotherapy, radiotherapy or hormonal therapy was administered depending on the stage and histopathological subtype. The preferred chemotherapeutic drug regimen was Docetaxel and Gemcitabine. Radiotherapy was rendered in ways of External Beam Radiotherapy (EBRT) of 50 gray in 25 fractions or Brachytherapy. Letrozole, an aromatase inhibitor at a dose of 2.5 mg was the preferred hormonal drug. The appropriate adjuvant treatment was decided as per multidisciplinary tumour board discussions.

The data compilation included basic socio-demographic data and clinicopathological features such as tumour size and site, preoperative radiological findings, tumour markers such as serum CA-125, Lactate dehydrogenase (LDH), Carcinoembryonic antigen (CEA), CA 19-9 and hormone statuses such as Estrogen receptor (ER) and Progesterone receptor (PR) status on immunohistochemistry. The data regarding the modality of surgery, adjuvant treatment and details of recurrence also were gathered for analysis. The data were categorised according to the pathological subclasses as, ULMS, HGESS and LGESS. The variants such as AS, UUS, alveolar rhabdomyosarcoma (ARMS) and embryonal rhabdomyosarcoma (ERMS) were collectively categorised under a group named ‘OTHERS’ due to paucity of cases. The data were retrieved from the computerised data information system and case files from medical records department of our institute.

Statistical analysis was done using SPSS software version 23.0. Descriptive method using measures of dispersion was used to analyse the socio-demographic data. Survival outcome analysis was done using the Kaplan–Meier method. Univariate analysis was used to correlate the prognostic factors associated with overall survival (OS) and disease-free survival (DFS). A *p*-value of <0.05 was considered significant in the study.

## Results

Out of the 85 cases taken up for the study, there were 44 cases of ULMS, 25 cases of LGESS, 9 cases of HGESS and 7 cases under the OTHERS category. The OTHERS category included one case of AS with sarcomatous overgrowth (SO), two cases of AS without SO, two cases of UUS, one case of ERMS and one case of ARMS.

Clinical characteristics are described in Table 1. The mean age of presentation in our study was 50.8 years for ULMS, 45.5 years for LGESS, 48.1 years for HGESS and 49.6 years in the OTHERS group. The majority belonged to the less than 50 years age group in all the categories (62.4%) and were premenopausal (57.1%). Both the cases of UUS were postmenopausal. Abnormal uterine bleeding (AUB) was the foremost presenting symptom in majority of the groups (ULMS = 21.2%, LGESS = 15.3%, HGESS = 4.7%).

Ultrasonography was the major preoperative imaging modality identified in our study population (ULMS = 25.9%, LGESS = 16.5%, HGESS = 5.9%, OTHERS = 4.7%).

CA-125 levels were available only in 48 cases (56.5%) (25 cases of ULMS, 13 cases of LGESS, 6 cases of HGESS and 4 cases of OTHERS category) and was normal in majority (ULMS = 21.2%, LGESS = 11.8%, HGESS = 4.7%, OTHERS = 4.7%). LDH levels were available in 45 cases (52.9%) which included 26 cases of ULMS, 13 cases of LGESS, 4 cases of HGESS and 2 cases of OTHERS category. The majority had normal LDH levels except in the OTHERS category where LDH which was available in only two of the cases were abnormal (ULMS = 20%, LGESS = 12.9%, HGESS = 3.5%; OTHERS = 2.4%). CEA levels were available in 20 cases (23.5%) which included 11 cases of ULMS, 6 cases of LGESS and 3 cases of HGESS. All the cases had normal CEA levels. CA 19-9 levels were available in 12 cases (14.1%) which included 5 cases of ULMS, 4 cases of LGESS and 3 cases of HGESS. All the cases had normal CA 19-9 levels except for 1 case of LGESS which showed abnormal value.

Pathological characteristics are described in Table 2. The majority of cases were predominantly diagnosed in stage I (ULMS = 37.6%, LGESS = 16.5%, HGESS = 4.7%; OTHERS = 4.7%) followed by stage II (ULMS = 10.6%, LGESS = 9.4%, HGESS = 3.5%; OTHERS = 1.2%). Stage III presentation was seen in two (2.4%) cases each of ULMS and LGESS and one (1.2%) case each of HGESS and OTHERS category. There was one (1.2%) case each of stage IV in all the categories.

Tumour size of more than 5 cm was noted in all the subclasses except LGESS where the tumour size of less 5 cm was seen (ULMS = 50.6%, HGESS = 7%, OTHERS = 8.2%; LGESS = 18.8%,). An intramurally located tumour was noted in ULMS (36.5%) and HGESS (7%). Subserosal tumours were seen in LGESS (15.3%) and OTHERS (5.9%) categories.

The ER and PR status were available only for the cases of LGESS and HGESS, 17 and 5 cases, respectively. Both the hormone status was positive in 11 (12.9%) cases of LGESS and 2 (2.4%) cases of HGESS. ER alone was positive in 5 (5.9%) and 1 (1.2%) LGESS and HGESS cases, respectively. PR alone was positive in one (1.2%) case each of LGESS and HGESS, respectively.

## Treatment

Surgical management with TAH with BSO was the primary mode of surgery in the majority in all the categories (ULMS = 31.8%, LGESS = 25.9%, HGESS = 5.9%; OTHERS = 4.7%). LND was done in 5 (5.9%) cases of ULMS, 1 (1.2%) case of LGESS and 2 (2.4%) cases each in HGESS and OTHERS categories. Omentectomy was done in six (7%) of ULMS, two (2.4%) cases of LGESS, four (4.7%) cases of HGESS and one (1.2%) case of OTHERS group. Adjuvant treatment was administered in 36 (42.5%) cases (Table 3). Radiotherapy in the form of Brachytherapy (8.2%) and EBRT (4.7%) were the major form rendered in ULMS. Hormonal treatment with letrozole was principally administered in LGESS (10.6%) and OTHERS category (2.4%). Chemotherapy with Docetaxel and Gemcitabine regimen was preferably administered in majority in HGESS (5.9%) (Table 4).

The median follow-up time was 37 months (Range 4–182 months). The overall recurrence rate in our study was 56.5%. To subclassify, it was seen in 28 (33%) cases of ULMS, 9 (36%) cases of LGESS, 7 (77.8%) cases of HGESS and 4 cases of the OTHERS category. AS patients had no recurrence.

**Figure 1.**
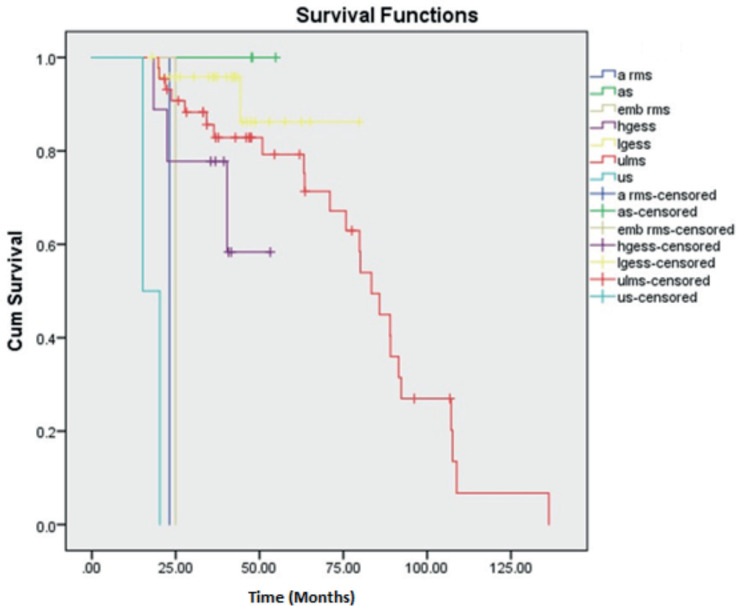
Kaplan Meier curve showing OS.

**Figure 2.**
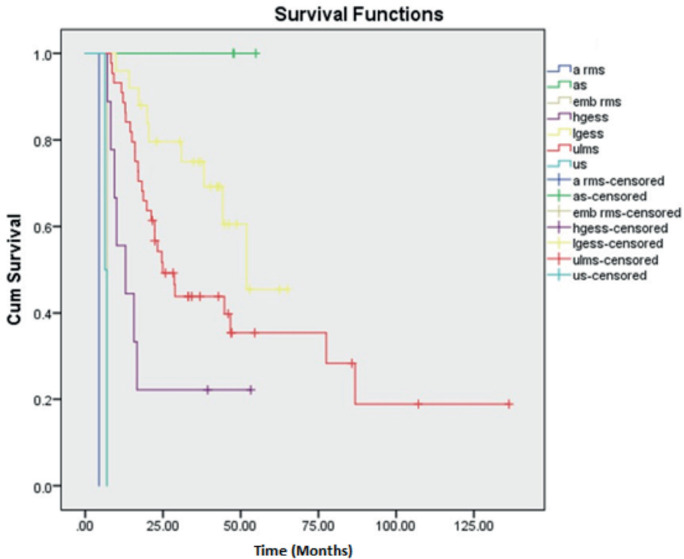
Kaplan Meier curve showing DFS.

There were three cases of AS of which two belonged to stage I and the other stage II. All three cases did not have a recurrence and are surviving till date. Thus, deriving a 5-year OS and DFS rate of 100%, respectively. Second, LGESS showed a better 5-year OS and DFS rate of 86.3% and 60.5%, respectively. The 5-year OS and DFS rates of ULMS were calculated to be 77.7% and 39.9%, respectively. The maximum follow-up of HGESS cases was 53 months (4.4 years). Hence the 3-year OS and DFS were calculated with values of 58.3% and 22.2%, respectively. The single cases of ERMS and ARMS in our study were of stage III and I, respectively. Both the cases had recurrence at 7 and 9 months, respectively. The two cases of UUS were of stage II and IV with recurrence at 11 and 6 months, respectively. All the above cases under the OTHERS category had succumbed to death depicting them as aggressive varieties. Kaplan Meier curves depicting OS and DFS are shown in Figures 1 and 2.

**Figure 3.**
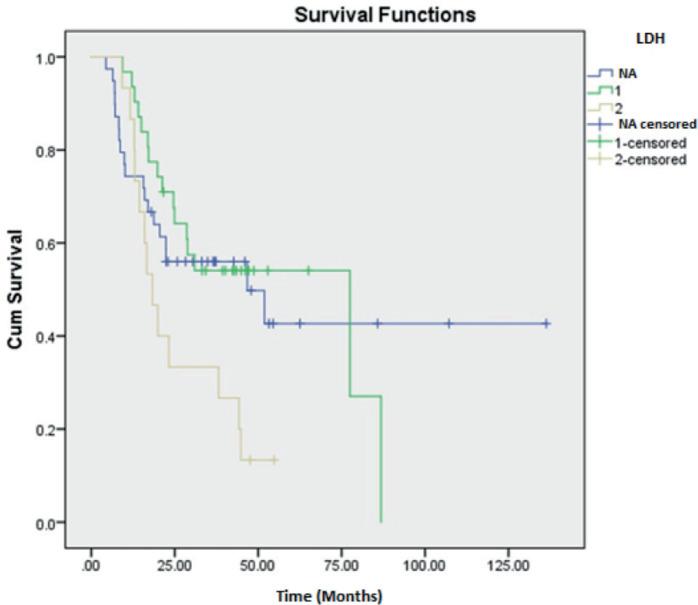
Kaplan Meier curve showing the effect of LDH value on recurrence.

**Figure 4.**
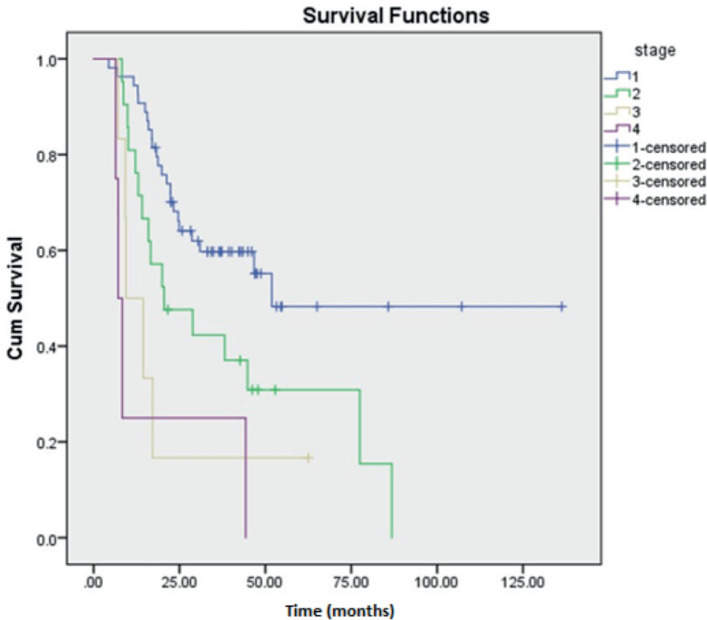
Kaplan Meier curve showing the effect of stage of the tumour on recurrence.

Prognostic variables on DFS and OS were studied using univariate analysis. Tumour size was found to be a significant factor affecting DFS of HGESS and OS of LGESS. LDH value was revealed to be affecting both DFS and OS of tumours under the OTHERS category. Stage of the tumour significantly affected the DFS and OS of LGESS, DFS of ULMS and OS of HGESS. The correlation of various clinicopathological factors affecting recurrence has revealed the significance of LDH value, the stage of the tumour and the nature of the adjuvant treatment (Table 5). Kaplan Meier curves demonstrating the clinicopathological features of LDH and stage of tumour affecting recurrence are shown in Figures 3 and 4.

## Discussion

USs are rare gynaecological malignancies with histological diversities and higher recurrence rate therefore leading to inferior survival outcomes [2]. This 10-year retrospective study aimed to provide an understanding of the clinicopathological features and its associated prognostic factors.

ULMS was the common histopathological subtype (51.8%) identified in our study consistent with the previous reports [2, 8]. The age of presentation which varies for different histologies. In our study, the mean age of ULMS was 50.8 years. ESS cases showed a mean age of 45.5 and 48.1 years for low-grade and high-grade, respectively. These are similar to the results of the study by Wang *et al* [9]. Most of the patients in our study were premenopausal except for AS and UUS which were mostly postmenopausal. UUS cases had the highest average age of presentation. This is consistent with a multi-centric study by Li *et al* [10] where the cases were predominantly premenopausal and UUS cases had the oldest age of presentation.

AUB was the commonest presenting symptom, with PMB was seen in UUS and AS. Mass per abdomen and abdominal pain were the other significant symptoms noted, concordant with the findings by Thangappah *et al* [11]. The symptoms are probably due to the rapid-growing nature of these tumours. Hence any form of genital bleeding and rapidly enlarging masses should prompt suspicion of US. Preoperative endometrial sampling could aid in the diagnosis [12]. 12.9% of the cases of our study were preoperatively diagnosed by endometrial biopsy.

Ultrasound was the major imaging modality. Although recent literature suggests Magnetic resonance imaging with features such as irregular margins and inhomogeneous architecture with higher T2 signals are necessary in the workup [13, 14]. Cost remains a limiting factor in its implementation especially in financially low-performing nations.

The diagnostic accuracy of the tumour markers CA-125, LDH, CEA and CA 19-9 were assessed. CA-125 lacked significance, aligning with the findings by Yilmaz *et al* [15], though Duk *et al* [16] linked elevated levels to be a poor prognosis. Stimulated mesothelial cells are known to cause elevated CA-125 in US. LDH emerged as a significant prognostic variable for recurrence with Song *et al* [17] highlighting its relevance in ULMS. Our study showed significance of LDH for the tumours such AS, UUS and RMS in addition to ULMS. There are few case reports showing exceedingly higher LDH values in RMS cases although their significance has not been reported [18, 19]. As LDH is an enzyme involved in metabolism of the cancer cells, it could be elevated in many other conditions and cannot considered as a specific marker [20]. DiSaia *et al* [21] identified elevated CEA in a few cases of US. However, it was not found significant. The role of CA 19-9 secreted by the epithelial cells of uterus has only a minimal role in gynaecological malignancies [22]. This proclaims that none of the tumour markers are specific or sensitive for US.

ER and PR expression was highly demonstrated in LGESS compared to HGESS and ULMS. Davidson *et al* [23] reported ER and PR expression in 53% and 67% of the LGESS, 31% and 47% of HGESS and 45% and 65% of ULMS cases, respectively. This makes hormonal therapy a better adjuvant modality for LGESS [24]. Apart from ER and PR literatures suggest several other molecular markers for prognostication. These include JAZF1-SUZ12 gene fusion which is present in significant proportion of LGESS cases. This is associated with better outcome. CD10 is also a useful diagnostic marker of LGESS. YWHAE-NUTM2A/B fusion is a hallmark for HGESS. This along with BCOR alterations are associated with aggressive tumour behaviour. Ki-67, a proliferation marker is highly expressed in HGESS [5, 25]. Overexpression of MCM2 is often observed in ULMS and it helps to differentiate from leiomyomas [26]. Androgen receptor is used as a prognostic marker for ULMS [27]. Although these biomarkers have not been analysed in our study.

Most of the cases had presented at an earlier stage of stage I and II. Survival analysis showed the significance of tumour stage on DFS of ULMS and LGESS and OS of LGESS and HGESS comparable with the study by Cheng *et al* [28]. ULMS showed a little higher OS and an equivalent DFS as that of Ayhan *et al* [29] reporting 54% and 42%, respectively. LGESS outcomes were lower than Zhou *et al* [30] demonstrating a 5-year OS and DFS of 96.7% and 91.8%, respectively. A previous study reported a 3-year OS of 27.3% for HGESS which is much lower than our study [10]. A study on AS by Mancari *et al* [31] revealed a 5-year OS and DFS to be 89.5% and 85.3%, respectively, which is lower than in our study. All the differences might be due to the differences in the patient cohort and treatment modalities. LGESS and AS showed favourable survival outcomes in comparison with UUS and HGESS exhibiting poor survival outcomes. These are supported by a few previous studies [2, 9–11].

TAH with BSO was the primary surgical approach. The role of BSO in cases of ULMS remains controversial. Analysis by a few survival studies analysing the effect of ovarian preservation on survival revealed nil significance [32, 33]. Nevertheless, BSO is recommended for ESS cases as they are hormone-responsive tumours [6].

The evidence regarding the mode of adjuvant therapies in US is limited. Hao *et al* [34] studied 2,897 cases of ULMS and reported better survival in the radiotherapy group. This supports the findings of our study. Studies to evaluate the role of AI in LGESS suggest AI offer prolonged disease-free periods even in metastatic settings [35]. A case report by Altal *et al* [36] demonstrated complete remission of advanced LGESS with hormonal therapy with letrozole at the dose of 2.5 mg per day similar to the dosage followed in our study. Zhang *et al* [37] reported the benefits of the chemotherapeutic regimen adopted for HGESS identical to our study. However, studies do suggest Doxorubicin-based regimens as the preferred regimen in the treatment of US [38, 39]. In recurrent cases of ULMS, Trabectidin has shown efficacy [40]. Targeted therapies are recently becoming an interest particularly for tumours with high tumour mutational burden. Pembrolizumab, immune checkpoint inhibitor have been proven effective in microsatellite instability-high ULMS [41]. Pazopanib is another approved drug for US [42].

Recurrence remains a key concern though our study’s rate was lower than the 53%–71% reported by Giuntoli *et al* [43], likely due to larger patient cohort in earlier stages than the advanced stages. The relapse rate of ESS ranges from 36% to 56% coinciding with that of LGESS. However, an increased recurrence rate was noted in HGESS in our study [44]. AS are reported to show a long latent course with good survival which is comparable with the cases of our study [45]. Tumour stage and size influenced survival and recurrence [2].

US is a rare tumour with a larger number of only retrospective studies in literature [2, 9–11]. Further prospective studies are essential to develop a definitive management protocol. The advantage of our study is that, this is an oncology-dedicated tertiary-care centre study. However, there are certain limitations which include its retrospective nature with smaller number of cases of subtypes like AS, UUS and RMS from a single centre. This led to the inability of a comprehensive subgroup analysis of all varieties separately.

## Conclusion

US tends to recur with prognosis varying among the subtypes. Our study revealed that AS and LGESS show better survival outcomes. Prognostic factors for recurrence were LDH value, stage of the tumour and nature of the adjuvant treatment. A prompt diagnosis at an earlier stage with a proper workup and timely intervention including its adjuvant treatment would help to mitigate the severity of the disease. A vigilant follow-up is recommended to pick up the recurrences beforehand.

## Conflicts of interest

None.

## Funding

No funding was received for the study.

## Author contributions

All the authors contributed to the conception of the work, preparation of manuscript and revision of the manuscript critically for intellectual content.

## Figures and Tables

**Table 1.** Clinicopathologic characteristics of study population.

Parameters			ULMS (n = 44)		LGESS (n = 25)		HGESS (n = 9)		OTHERS (n = 7)	
Age	<50 years		25 (29.4)		19 (22.4)		5 (5.9)		4 (4.7)	
	>50 years		19 (22.3)		6 (7.1)		4 (4.7)		3 (3.5)	
Parity	Nulliparous		2 (2.4)		2 (2.4)		0 (0)		1 (1.2)	
	Primiparous		1 (1.2)		2 (2.4)		1 (1.2)		1 (1.2)	
	Multiparous		41 (48.2)		21 (24.7)		8 (9.4)		5 (5.9)	
Symptoms	AUB		18 (21.2)		13 (15.3)		4 (4.7)		3 (3.5)	
	Mass		10 (11.8)		3 (3.5)		3 (3.5)		1 (1.2)	
	Pain		8 (9.4)		9 (10.6)		2 (2.4)		1 (1.2)	
	PMB		5 (5.9)		4 (4.7)		2 (2.4)		4 (4.7)	
Menopausal status	Premenopausal		27 (31.2)		17 (20)		5 (5.9)		3 (3.5)	
	Postmenopausal		17 (20)		8 (9.4)		4 (4.7)		4 (4.7)	
Imaging	USG		22 (25.9)		14 (16.5)		5 (5.9)		4 (4.7)	
	CT		15 (17.6)		8 (9.4)		3 (3.5)		3 (3.5)	
	MRI		7 (8.2)		3 (3.5)		1 (1.2)		0 (0)	
Endometrial biopsy	Normal		8 (9.4)		5 (5.9)		1 (1.2)		0 (0)	
	Abnormal		1 (1.2)		7 (8.2)		2 (2.4)		1 (1.2)	
	Unavailable		35 (41.2)		13 (15.3)		6 (7.1)		6 (7.1)	
Tumor markers	CA-125	Normal		18 (21.2)		10 (11.8)		4 (4.7)		4 (4.7)
		Abnormal		7 (8.2)		3 (3.5)		2 (2.4)		0 (0)
		Unavailable		19 (22.4)		12 (14.1)		3 (3.5)		3 (3.5)
	LDH	Normal		17 (20)		11 (12.9)		3 (3.5)		0 (0)
		Abnormal		9 (10.6)		3 (3.5)		1 (1.2)		2 (2.4)
		Unavailable		18 (21.2)		11 (12.9)		5 (5.9)		5 (5.9)
	CEA	Normal		11 (12.9)		6 (7)		3 (3.5)		0 (0)
		Abnormal		0 (0)		0 (0)		0 (0)		0 (0)
		Unavailable		33 (38.8)		19 (22.4)		6 (7)		7 (8.2)
	CA 19-9	Normal		5 (5.9)		3 (3.5)		3 (3.5)		0 (0)
		Abnormal		0 (0)		1 (1.2)		0 (0)		0 (0)
		Unavailable		39 (45.9)		21 (24.7)		6 (7)		6 (7)

CEA, carcinoembryonic antigen; HGESS, high grade endometrial stromal sarcoma; LGESS, low grade endometrial stromal sarcoma; LDH, lactate dehydrogenase; ULMS, uterine leiomyosarcoma

**Table 2.** Details of final histopathological examination report.

Parameters		ULMS (*n* = 44)	LGESS (*n* = 25)	HGESS (*n* = 9)	OTHERS (*n* = 7)
**Stage**	I	32 (37.6)	14 (16.5)	4 (4.7)	4 (4.7)
	II	9 (10.6)	8 (9.4)	3 (3.5)	1 (1.2)
	III	2 (2.4)	2 (2.4)	1 (1.2)	1 (1.2)
	IV	1 (1.2)	1 (1.2)	1 (1.2)	1 (1.2)
**Tumor size**	<5 cm	1 (1.2)	16 (18.8)	3 (3.5)	0 (0)
	>5 cm	43 (50.6)	9 (10.6)	6 (7)	7 (8.2)
**Tumor site**	Intramural	31 (36.5)	10 (11.8)	6 (7)	2 (2.4)
	Subserosal	8 (9.4)	13 (15.3)	2 (2.4)	5 (5.9)
	Submucosal	5 (5.9)	2 (2.4)	1 (1.2)	0 (0)
**IHC**	ER+ PR−	0 (0)	5 (5.9)	1 (1.2)	0 (0)
	ER− PR+	0 (0)	1 (1.2)	1 (1.2)	0 (0)
	Both+	0 (0)	11 (12.9)	2 (2.4)	0 (0)
	Both−	0 (0)	0 (0)	1 (1.2)	0 (0)
	Not available	44 (51.8)	8 (9.4)	4 (4.7)	7 (8.2)
**Other structures involvement**	Ovary	6 (7.1)	5 (5.9)	1 (1.2)	1 (1.2)
	Lymph node	1 (1.2)	1 (1.2)	2 (2.4)	1 (1.2)

ER, estrogen receptor; HGESS, high grade endometrial stromal sarcoma; LGESS, low grade endometrial stromal sarcoma; PR, progesterone receptor

**Table 3.** Type of surgeries done in study population.

Surgery	ULMS (*n* = 44)	LGESS (*n* = 25)	HGESS (*n* = 9)	OTHERS (*n* = 7)
**TAH**	7 (8.2)	1 (1.2)	0 (0)	1 (1.2)
**TAH + BSO**	27 (31.8)	22 (25.9)	5 (5.9)	4 (4.7)
**TAH + BSO + LND**	4 (4.7)	0 (0)	0 (0)	1 (1.2)
**TAH + BSO + OMENTECTOMY**	5 (5.9)	1 (1.2)	2 (2.4)	0 (0)
**TAH + BSO + LND + OMENTECTOMY**	1 (1.2)	1 (1.2)	2 (2.4)	1 (1.2)

BSO, bilateral salpingoophorectomy; HGESS, high grade endometrial stromal sarcoma; LGESS, low grade endometrial stromal sarcoma; LND, lymph node dissection; TAH, total abdominal hysterectomy

**Table 4.** Adjuvant treatment administered post-surgery.

Surgery		ULMS (*n* = 44)	LGESS (*n* = 25)	HGESS (*n* = 9)	OTHERS (*n* = 7)
**Chemotherapy**		3 (3.5)	0 (0)	5 (5.9)	1 (1.2)
**Radiotherapy**	EBRT	4 (4.7)	0 (0)	0 (0)	0 (0)
	Brachytherapy	7 (8.2)	1 (1.2)	1 (1.2)	1 (1.2)
**Hormonal therapy**		0 (0)	9 (10.6)	0 (0)	2 (2.4)
**Chemotherapy+ EBRT**		1 (1.2)	0 (0)	0 (0)	0 (0)
**Chemotherapy+ Hormonal therapy**		0 (0)	1 (1.2)	0 (0)	0 (0)
**None**		29 (34.1)	14 (16.5)	3 (3.5)	3 (3.5)

EBRT, external beam radiotherapy; HGESS, high grade endometrial stromal sarcoma; LGESS, low grade endometrial stromal sarcoma; ULMS, uterine leiomyosarcoma

**Table 5.** Correlation of recurrence with clinicopathological features.

Parameters				ULMS (*n* = 28)	LGESS (*n* = 9)	HGESS (*n* = 7)	OTHERS (*n* = 4)	*p*-value
**Age**	<50 years			18 (40.9)	7 (28)	5 (55.6)	3 (42.9)	0.17
	>50 years			10 (22.7)	2 (8)	2 (22.2)	1 (14.3)	
**Menopausal status**	Premenopausal			18 (40.9)	7 (28)	5 (55.6)	2 (28.6)	0.24
	Postmenopausal			10 (22.7)	2 (8)	2 (22.2)	2 (28.6)	
**Tumor size**	<5 cm			0 (0)	4 (16)	1 (11.1)	0 (0)	0.06
	>5 cm			28 (63.6)	5 (20)	6 (66.7)	4 (57.1)	
**Tumor markers**	CA-125	Normal		13 (29.5)	1 (4)	2 (22.2)	2 (28.6)	0.12
		Abnormal		6 (13.6)	2 (8)	2 (22.2)	0 (0)	
		Unavailable		9 (20.5)	6 (24)	3 (33.3)	2 (28.6)	
	LDH	Normal		10 (22.7)	3 (12)	2 (22.2)	1 (14.3)	0.03
		Abnormal		9 (20.5)	3 (12)	1 (11.1)	0 (0)	
		Unavailable		9 (20.5)	3 (12)	4 (44.4)	3 (42.9)	
**Hormone status**	ER+ PR−			0 (0)	1 (4)	1 (11.1)	0 (0)	0.37
	ER− PR+			0 (0)	1 (4)	1 (11.1)	0 (0)	
	Both+			0 (0)	5 (20)	1 (11.1)	0 (0)	
	Both−			0 (0)	0 (0)	1 (11.1)	0 (0)	
	Not available			28 (63.6)	2 (8)	3 (33.3)	4 (57.1)	
**Stage**	I			17 (38.6)	2 (8)	2 (22.2)	2 (28.6)	0.01
	II			8 (18.2)	5 (20)	3 (33.3)	0 (0)	
	III			2 (4.5)	1 (4)	1 (11.1)	1 (14.3)	
	IV			1 (2.3)	1 (4)	1 (11.1)	1 (14.3)	
**Type of surgery**	TAH			4 (9.1)	1 (4)	0 (0)	1 (14.3)	0.19
	TAH + BSO			18 (40.1)	8 (32)	3 (33.3)	2 (28.6)	
	TAH + BSO + LND			2 (4.5)	0 (0)	0 (0)	1 (14.3)	
	TAH + BSO + OMENTECTOMY			3 (6.8)	0 (0)	0 (0)	0 (0)	
	TAH + BSO + LND + OMENTECTOMY			1(2.3)	0 (0)	0 (0)	0 (0)	
**Adjuvant treatment**	Chemotherapy			3 (6.8)	0 (0)	5 (55.6)	0 (0)	0.01
	Radiotherapy		EBRT	3 (6.8)	0 (0)	0 (0)	0 (0)	
			Brachytherapy	1 (2.3)	0 (0)	0 (0)	0 (0)	
	Hormonal therapy			0 (0)	2 (8)	0 (0)	2 (28.6)	
	Chemotherapy+ EBRT			1 (2.3)	0 (0)	0 (0)	0 (0)	
	Chemotherapy+ Hormonal therapy			0 (0)	1 (4)	0 (0)	0 (0)	
	None			20 (45.5)	6 (24)	2 (22.2)	2 (28.6)	

BSO, Bilateral Salpingoophorectomy; EBRT, external beam radiotherapy; ER, estrogen receptor; HGESS, high grade endometrial stromal sarcoma; LGESS, low grade endometrial stromal sarcoma; LND, Lymph Node Dissection; PR, progesterone receptor; TAH, total abdominal hysterectomy; ULMS, uterine leiomyosarcoma

## References

[1] Giannini A, Golia D'augè T, Bogani G (2023). Uterine sarcomas: a critical review of the literature. Eur J Obstet Gynecol Reprod Biol.

[2] Wang F, Dai X, Chen H (2022). Clinical characteristics and prognosis analysis of uterine sarcoma: a single-institution retrospective study. BMC Cancer.

[3] Skorstad M, Kent A, Lieng M (2016). Uterine leiomyosarcoma – incidence, treatment, and the impact of morcellation. A nationwide cohort study. Acta Obstet Gynecol Scand.

[4] Harlow BL, Weiss NS, Lofton S (1986). The epidemiology of sarcomas of the uterus. J Natl Cancer Inst.

[5] Ferrandina G, Aristei C, Biondetti PR (2020). Italian consensus conference on management of uterine sarcomas on behalf of S.I.G.O. (Societa’ italiana di Ginecologia E Ostetricia). Eur J Cancer.

[6] Mbatani N, Olawaiye AB, Prat J (2018). Uterine sarcomas. Int J Gynecol Obstet.

[7] Cantú De León D, González H, Pérez Montiel D (2013). Uterine sarcomas: review of 26 years at The Instituto Nacional de Cancerologia of Mexico. Int J Surg.

[8] Barquet-Muñoz SA, Isla-Ortiz D, Montalvo-Esquivel G (2019). Prognostic factors associated with uterine sarcomas: the experience of a single institution. J Obstet Gynaecol J Inst Obstet Gynaecol.

[9] Wang JF, Li C, Yang JY (2024). Clinicopathological characteristics and prognosis of uterine sarcoma: a 10-year retrospective single-center study in China. Diagn Pathol.

[10] Li D, Yin N, Du G (2020). A real-world study on diagnosis and treatment of uterine sarcoma in western China. Int J Biol Sci.

[11] Thangappah RBP (2019). Uterine sarcoma: a clinico-pathological study. J Obstet Gynaecol India.

[12] Dijkhuizen FPHLJ, Mol BWJ, Br�Lmann HAM (2000). The accuracy of endometrial sampling in the diagnosis of patients with endometrial carcinoma and hyperplasia: a meta-analysis. Cancer.

[13] Hindman N, Kang S, Fournier L (2023). MRI evaluation of uterine masses for risk of leiomyosarcoma: a consensus statement. Radiology.

[14] Valletta R, Corato V, Lombardo F (2024). Leiomyoma or sarcoma? MRI performance in the differential diagnosis of sonographically suspicious uterine masses. Eur J Radiol.

[15] Yilmaz N, Sahin I, Kilic S (2009). Assessment of the predictivity of preoperative serum CA 125 in the differential diagnosis of uterine leiomyoma and uterine sarcoma in the Turkish female population. Eur J Gynaecol Oncol.

[16] Duk JM, Bouma J, Burger GTN (1994). CA 125 in serum and tumor from patients with uterine sarcoma. Int J Gynecol Cancer.

[17] Song K, Yu X, Lv T (2018). Expression and prognostic value of lactate dehydrogenase-A and -D subunits in human uterine myoma and uterine sarcoma. Med (Baltimore).

[18] Tamura S, Hayashi T, Ichimura T (2022). Characteristic of uterine rhabdomyosarcoma by algorithm of potential biomarkers for uterine mesenchymal tumor. Curr Oncol.

[19] Li ZJ, Li CL, Wang W (2021). Diagnosis and treatment of pleomorphic rhabdomyosarcoma of the uterus: a rare case report and review of the literature. J Int Med Res.

[20] Suh DS, Song YJ, Roh HJ (2021). Preoperative blood inflammatory markers for the differentiation of uterine leiomyosarcoma from leiomyoma. Cancer Manag Res.

[21] Disaia PJ, Haverback BJ, Dyce BJ (1975). Carcinoembryonic antigen in patients with gynecologic malignancies. Am J Obstet Gynecol.

[22] Lee T, Teng TZJ, Shelat VG (2020). Carbohydrate antigen 19–9 — tumor marker: past, present, and future. World J Gastrointest Surg.

[23] Davidson B, Kjæreng ML, Førsund M (2016). Progesterone receptor expression is an independent prognosticator in FIGO stage I uterine leiomyosarcoma. Am J Clin Pathol.

[24] Maccaroni E, Lunerti V, Agostinelli V (2022). New insights into hormonal therapies in uterine sarcomas. Cancers.

[25] Pérez-Fidalgo JA, Ortega E, Ponce J (2023). Uterine sarcomas: clinical practice guidelines for diagnosis, treatment, and follow-up, by Spanish group for research on sarcomas (GEIS). Ther Adv Med Oncol.

[26] Guo J, Zheng J, Tong J (2024). Potential markers to differentiate uterine leiomyosarcomas from leiomyomas. Int J Med Sci.

[27] Baek MH, Park JY, Park Y (2018). Androgen receptor as a prognostic biomarker and therapeutic target in uterine leiomyosarcoma. J Gynecol Oncol.

[28] Cheng G, Li Y, Qu P (2020). Clinicopathological characteristics of patients with uterine sarcoma: clinical presentation, treatment, and survival outcomes. Int J Clin Exp Med.

[29] Ayhan A, Gungorduk K, Khatib G (2021). Prognostic factors and survival outcomes of women with uterine leiomyosarcoma: a Turkish Uterine Sarcoma Group Study-003. Curr Probl Cancer.

[30] Zhou J, Zheng H, Wu SG (2015). Influence of different treatment modalities on survival of patients with low-grade endometrial stromal sarcoma: a retrospective cohort study. Int J Surg.

[31] Mancari R, Yusuf Y, Macuks R (2024). Prognostic factors in uterine adenosarcoma: subanalysis of the SARCUT study. Front Oncol.

[32] Giuntoli RL, Metzinger DS, Dimarco CS (2003). Retrospective review of 208 patients with leiomyosarcoma of the uterus: prognostic indicators, surgical management, and adjuvant therapy. Gynecol Oncol.

[33] Gadducci A, Landoni F, Sartori E (1996). Uterine Leiomyosarcoma: analysis of Treatment Failures and Survival. Gynecol Oncol.

[34] Hao Z, Yang S (2022). The role of postoperative radiotherapy in patients with uterine sarcomas: a PSM-IPTW analysis based on SEER database. Front Surg.

[35] Crowley F, Cadoo KA, Chiang S (2022). Evaluating the role of aromatase inhibitors in the treatment of low-grade endometrial stromal sarcomas. Gynecol Oncol Rep.

[36] Altal OF, Al Sharie AH, Halalsheh OM (2021). Complete remission of advanced low-grade endometrial stromal sarcoma after aromatase inhibitor therapy: a case report. J Med Case Rep.

[37] Zhang Y, Chen C, Ren M (2019). Treatment of uterine high-grade endometrial stromal sarcoma with apatinib combined with chemotherapy. Med (Baltimore).

[38] Turinetto M, Meeus P, Ray-Coquard I (2023). Doxorubicin combined with Trabectedin in systemic first-line M+/recurrent leiomyosarcoma patients. Curr Opin Oncol.

[39] Kim T, Hao C, Pan M (2025). Gemcitabine plus docetaxel, dacarbazine, doxorubicin combinations, or doxorubicin alone as first-line treatment for advanced/metastatic leiomyosarcoma: a retrospective analysis at a sarcoma center. Diseases.

[40] Monk BJ, Blessing JA, Street DG (2012). A phase II evaluation of trabectedin in the treatment of advanced, persistent, or recurrent uterine leiomyosarcoma: a gynecologic oncology group study. Gynecol Oncol.

[41] Wang YJ, Williams HR, Brzezinska BN (2021). Use of pembrolizumab in MSI-high uterine leiomyosarcoma; a case report and review of the literature. Gynecol Oncol Rep.

[42] Cuppens T, Tuyaerts S, Amant F (2015). Potential therapeutic targets in uterine sarcomas. Sarcoma.

[43] Giuntoli RL, Garrett-Mayer E, Bristow RE (2007). Secondary cytoreduction in the management of recurrent uterine leiomyosarcoma. Gynecol Oncol.

[44] Amant F, Coosemans A, Debiec-Rychter M (2009). Clinical management of uterine sarcomas. Lancet Oncol.

[45] Korets SB, Curtin JP (2012). Surgical options for recurrent uterine sarcomas. Am Soc Clin Oncol Educ Book.

